# Nitric Oxide-Donor SNAP Induces *Xenopus* Eggs Activation

**DOI:** 10.1371/journal.pone.0041509

**Published:** 2012-07-23

**Authors:** Michal Jeseta, Matthieu Marin, Hana Tichovska, Petra Melicharova, Katia Cailliau-Maggio, Alain Martoriati, Arlette Lescuyer-Rousseau, Rémy Beaujois, Jaroslav Petr, Marketa Sedmikova, Jean-François Bodart

**Affiliations:** 1 Veterinary Research Institute, Department of Genetics and Reproduction, Brno, Czech Republic; 2 Czech University of Life Sciences in Prague, Department of Veterinary Science, Prague, Czech Republic; 3 Université Lille1, Sciences et Technologies, Laboratoire de Régulation des Signaux de Division - EA 4479, Villeneuve d’Ascq, France; 4 Interdisciplinary Research Institute USR 3078 CNRS, Villeneuve d‘Ascq, France; 5 Research Institute of Animal Production, Prague, Czech Republic; Institut Jacques Monod, France

## Abstract

Nitric oxide (NO) is identified as a signaling molecule involved in many cellular or physiological functions including meiotic maturation and parthenogenetic activation of mammalian oocytes. We observed that nitric oxide donor SNAP was potent to induce parthenogenetic activation in *Xenopus* eggs. NO-scavenger CPTIO impaired the effects of SNAP, providing evidence for the effects of the latter to be specific upon NO release. In *Xenopus* eggs, SNAP treatment induced pigment rearrangement, pronucleus formation and exocytosis of cortical granules. At a biochemical level, SNAP exposure lead to MAPK and Rsk inactivation within 30 minutes whereas MPF remained active, in contrast to calcium ionophore control where MPF activity dropped rapidly. MAPK inactivation could be correlated to pronuclear envelope reformation observed. In SNAP-treated eggs, a strong increase in intracellular calcium level was observed. NO effects were impaired in calcium-free or calcium limited medium, suggesting that that parthenogenetic activation of Xenopus oocytes with a NO donor was mainly calcium-dependent.

## Introduction

Nitric oxide (NO) is produced by Nitric Oxide Synthase (NOS) from L-Arginine and molecular oxygen, through a process that can also generate L-citruline [1]. NOS is expressed in three isoforms, which are homodimers [1], [2]. Constitutional isoforms of NOS, neuronal NOS (nNOS) and endothelial NOS (eNOS), are calcium and calmodulin-dependent and produce small amounts of NO for a short time lapse [2], [3]. An inducible isoform of NOS (iNOS) drives sustained NO production [3], [4], which is independent on calcium or calmodulin [5]. NOS isoforms have been detected in many cellular type [6]. Particularly, NOS have been isolated from a variety of mammalian reproductive tissues like ovary, uterus, testis or epididymis; the role of NO has been emphasized in many physiological processes including reproduction [3], [7]. NO was involved for example in the regulation of follicle growth and ovulation in mouse [8], spermatogenesis in human [9], embryo implantation in rats [7] and meiosis in pig and mouse [8], [10]. The data collected in the different above-mentioned species suggest that NO might have a widely role in reproduction through mechanisms conserved through evolution but one cannot discard that NO effects may be also dependent upon the species considered.

Meiosis is a mode of cell division in which a diploid cell undergoes through two successive divisions without replication, to produce haploid cells, namely oocytes and polar bodies in case of female gametes. Vertebrate oocyte is arrested in prophase of the first meiotic division (MI), resume meiosis in response to hormonal stimulation, in the process called maturation, and are stopped at metaphase of the second division, in anticipation to fertilization [11]. Metaphase II arrest is due to a cytostatic factor (CSF), whose function is to maintain high level of active MPF (M-Phase Promoting Factor ) within the cells. MPF promotes M-phase entry during mitosis or meiosis, and is made up of a catalytic subunit, Cdk1, and a regulatory sub-unit, Cyclin B [12]. The activity of this heterodimer is regulated by inhibitory phosphorylation on Thr14 and Tyr15, achieved by Wee1 and Myt1 kinases [13] and Cyclin B level, which can be degraded through the ubiquitin pathway [14]. Maintenance in metaphase II arrest is then achieved by preventing Cyclin B degradation. Though the nature of CSF has remained elusive for decades, it involved the Mitogen-Activated Protein Kinase (MAPK) cascade, whose components are involved in the prevention of Cyclin B degradation, and Emi2, which acts as an inhibitor of an Ubiquitin Ligase responsible from the metaphase-anaphase transition, the Anaphase Promoting Complex (APC) [15]–[17].

Maintenance of MPF activity in metaphase II-arrested oocytes, is broken by the interaction of sperm with the egg membrane, which leads to the activation of the egg metabolism and meiosis completion [18]. These cellular events, which are prepared by the cellular differentiation period related to maturation, also allow the transition from the eggs to the embryos [19]. They include the onset of the different polyspermy blocks, the completion of meiosis and the onset of embryonic mitosis [19]–[21]. Completion of meiosis is attested by the extrusion of the second polar body and pronucleus formation. Following fertilization as well as following parthenogenetic activation, MPF drops within five minutes while MAPK remains active for 30 minutes before decreasing [22], [23]. Inactivation of MPF is mainly due to the degragation of the Cyclin B whereas inactivation of MAPK reflects the degradation of its upstream activator Mos [24]. Exit from metaphase II could be mediated by a transient or sustained increase in intracellular calcium, depending on the considered species. In *Xenopus laevis*, the egg activation is mediated by a rise in intracellular calcium concentration with specialized spatial and temporal dynamics [19], and CaMKII is considered as the key protein in MPF down-regulation, through promoting Emi2 degradation and APC activation [25]. The specialized fertilization-specific calcium signal is shaped like a slow sweeping wave followed by a high calcium plateau that lasts for several minutes [26]–[28]. In non-excitable cells in general and in *Xenopus* oocyte particularly, an increase of intracellular calcium concentration could come both from release of calcium from internal stores (reticular or/and mitonchodrial stores) and influx from the external medium through calcium channels. In response to intracellular calcium stores depletion (*i.e.* rise of cytosolic calcium concentration), calcium enters the cells through calcium channels from the outside. The mechanism is called Store-Operated Calcium Entry (SOCE, through SOC channels) also known as capacitative calcium entry [29]. It is to note that in metaphase II-arrested oocytes SOCE could not be activated any more as a consequence of MPF activation [27], [29], [30]. Therefore, the calcium signalling pathways associated with oocyte egg activation is generated without any contribution of SOCE channels. Exit from metaphase II can be mimicked or stimulated by the calcium ionophore A23187 or by kinase inhibitors [22], [31]. As described and reviewed in [32], the maturation process implies numerous reorganizations of calcium signaling actors such as internalization of plasma membrane calcium ATPase (PMCA) or expression at the plasma membrane of calcium-activated chloride channels to prevent polyspermy.

**Table 1:** Effect of nitric oxide donor SNAP on parthenogenetic activation of matured oocytes (2 hours incubation).

Treatment	Number of oocytes(number of females)	% oocytes with pigment rearrangement	±SEM
Control	255 (19)	0^A^	0
SNAP 2.5 mM	312 (12)	40.04^B^	6.27
SNAP 5 mM	540 (17)	77.12^C^	3.02
A23187 10 µM	414 (19)	95.59^D^	1.03

A,B,C,D Statistically significant differences (P<0.05) in the ratio of activated oocytes are indicated by different superscripts.

**Figure 1:**
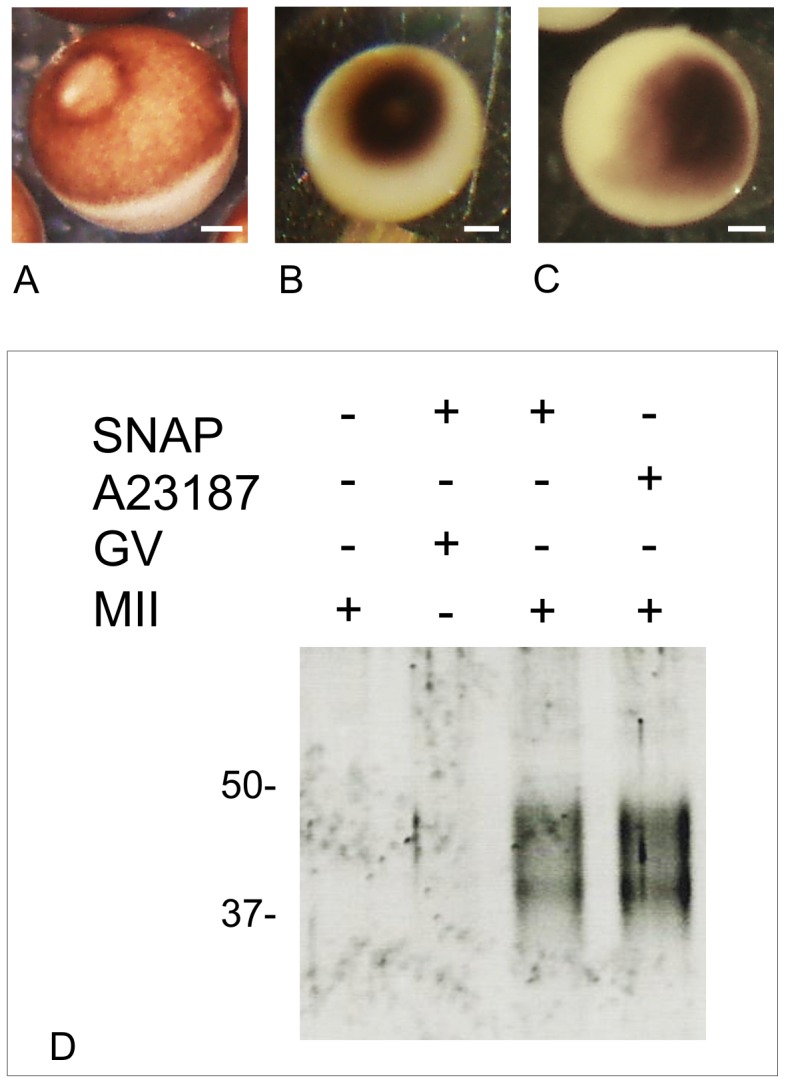
Nitric oxide donor SNAP induces cortical reaction typical of parthenogenetic activation in *Xenopus laevis* matured oocytes. (A) Typical morphologies of *Xenopus laevis* oocytes in control batches, arrested in metaphase II, (B) following parthenogenetic activation by A23187 treatment or (C) following SNAP treatment. Scale bars represent 200 µm. (D) SNAP treatment induces release of cortical granule lectins from *Xenopus* oocytes. Oocytes were incubated for 1 hour without or with 5 mM SNAP or 10 µM A23187. After 1 hour the fluid surrounding oocytes (15 µl) was collected for analysis by SDS-PAGE and SYPRO Ruby staining. Molecular weight standards are indicated in kDa.

Nitric oxide has been considered as a potential regulator of meiosis [8], [10]. Free radical nitric oxide was first reported to trigger parthenogenetic activation in sea urchin oocytes and suggested as a potential physiological regulator for egg activation [33]. This proposal has been shaded by the fact that nitric oxide did not appear as a key factor for fertilization in mice and ascidians. Sperm or sperm extract injections induce strong calcium response without affecting nitric oxide level [34]. Nevertheless, nitric oxide donors induce increase of intracellular levels of free calcium in mouse, ascidian [34] and sea urchin eggs [35], suggesting NO potentially acts through calcium mobilization. In porcine oocytes, NO donors were explored as potent parthenogenetic inducers [36], and egg activations were prevented by use of BAPTA-calcium chelator [37]. Then, NO is rather not a primary stimulus for oocyte activation, though it drives parthenogenesis through calcium mobilization in this mammalian model. However in porcine oocytes cumulus cells around oocyte play an important role in supporting maturation and the gap junctions between cumulus cells and oocyte enable transport of small molecules regulating meiotic maturation [38]. In previous reports were presented differences between DO (denuded oocytes) and COC (cumulus oocyte complexes) in various effect of NOS inhibitors on meiotic maturation in pig or mouse oocytes [10], [39]. Then, it appeared difficult to discriminate if the effects of NO donors are due to effects on follicular or on oocytes itselves.

Our current understanding of meiosis regulation in vertebrate oocytes largely benefited from studies performed in amphibian models such as *Xenopus laevis*. Oocytes from this animal model offer several advantages including year-around availability, cell cycle synchronicity and ease of amenability for manipulation and large amount of protein, enabling biochemical studies on 1/10^th^ of cells. Noticeably, oocytes resume meiosis under progesterone stimulation, in a process that is totally independent of follicular cells. Therefore, the present study aimed at evaluating the effect of nitric oxide donors on metaphase II block in *Xenopus laevis* oocytes. We tested the potential effects of NO donors on the activation of *Xenopus laevis* oocytes and thereby determine their effect on cortical exocytosis, cortical reaction, pronuclear formation. Here we report for the first time a parthenogenetic activation induced by NO donor in aquatic vertebrates - amphibian: nitric oxide donor - SNAP (S-nitroso-N-acetyl-DL-penicillamine) drives an atypical parthenogenetic activation of *Xenopus* eggs where MAPK cascade is broken in absence of MPF inactivation. Dependency upon Ca^2+^-dependent mechanisms is discussed.

**Table 2:** Effect of three different nitric oxide donors on parthenogenetic activation of *Xenopus laevis* matured oocytes.

Concentration of nitricoxide donor	Rate of activated oocytes (%) after 2 hours treatment with nitric oxide donor (±SEM)					
	n	SNAP	n	SNP	n	NOR5
0 mM	84	1,23 (±3,7)^A^	57	0 (±0,0)^A^	98	0 (±0,0)^A^
1 mM	91	11,66 (±11,85)^A^	67	8,07 (±8,78)^A^	106	3,3 (±5,77)^A^
2 mM	93	15,03 (±9,11)^A^	54	7,14 (±5,36)^A^	120	4,9 (±7,28)^A^
5 mM	162	80,14 (±9,78)^A^	39	3,03 (±4,29)^B^		not determined*
10 mM		not determined	49	8,33 (±1,67)		not determined*

(A–B) Values with different superscripts within the same row are significantly different.

* high concetration of NOR5 strongly increase rate of lytic oocytes.

Data are shown as mean percentage of activation of five independent experiments for each experimental group.

## Methods

### Ethics Statement

All animal experiments were performed at the animal facility of the USTL Lille according to the rules of the European Community Council guidelines (86/609/EEC) for laboratory animal experimentation. The animal protocol was approved by the local institutional review board (Comité d’Ethique en Expérimentation Animale Nord-Pas-De-Calais, CEEA 07/2010).

**Figure 2:**
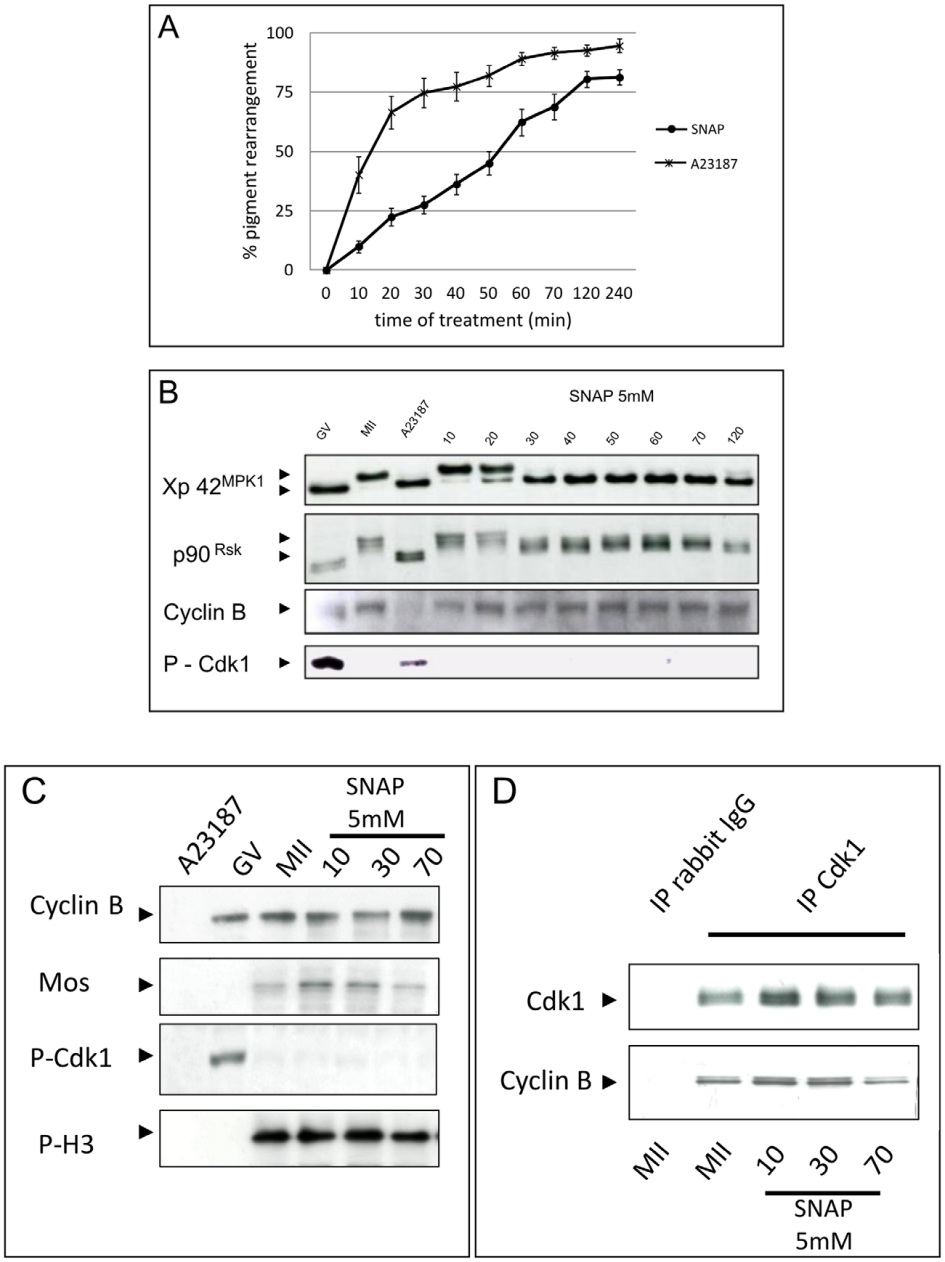
Kinetic of SNAP induced activation. (A) Kinetic of parthenogenetic activation after SNAP treatment. Matured oocytes were cultured with 5 mM SNAP or 10 µM A23187; cortical reaction was scored at 10 minutes time intervals. Data are shown as mean percentage of activation ± SEM of five independent experiments. (B) Western blot analysis. Each time 5 oocytes were taken off, homogenized in lysis buffer, and immunoblotted with antibodies against Cyclin B, P-Tyr15 Cdk1, Xp42^Mpk1^ and p90^Rsk^. (C) Western blot analysis. Neither Cyclin B or Mos were degraded in SNAP-treated matured oocytes (both were still detected 70 min after the beginning of the treatment); MPF activity was attested by its ability to phosphorylate histone H3. It is to note that Cdk1 remained unphosphorylated at tyrosine 15. (D) Immunoprecipitation. Cdk1 and Cyclin B are complexed together i metaphase II arrested oocytes. SNAP did not induce any separation between the two partners of the MPF heterodimer, even after 70 minutes.

### Handling of Frogs and Oocytes

After anesthetizing *Xenopus* females (purchased from the University of Rennes I, France) by immersion in 1 g/l MS222 solution (tricaine methane sulfonate; Sandoz), ovarian lobes were surgically removed and placed in ND96 medium (96 mM NaCl, 2 mM KCl, 1.8 mM CaCl_2_, 1 mM MgCl_2_, 5 mM Hepes–NaOH, pH 7.5). Fully grown stage VI oocytes were isolated and defolliculated by partial collagenase treatment for 30 min (1 mg/ml collagenase A, Roche Applied Science) followed by a manual microdissection. Oocytes were stored at 14°C in ND96 medium until experiments. Meiotic resumption was induced by incubation of oocytes at 19°C in ND96 medium containing 10 µM of progesterone (Sigma–Aldrich). Maturation process (or M-Phase entry) was scored by the appearance of a white spot at the animal pole of the oocyte. Activation was scored by occurence of pigment rearrangement typical of cortical reaction.

**Figure 3:**
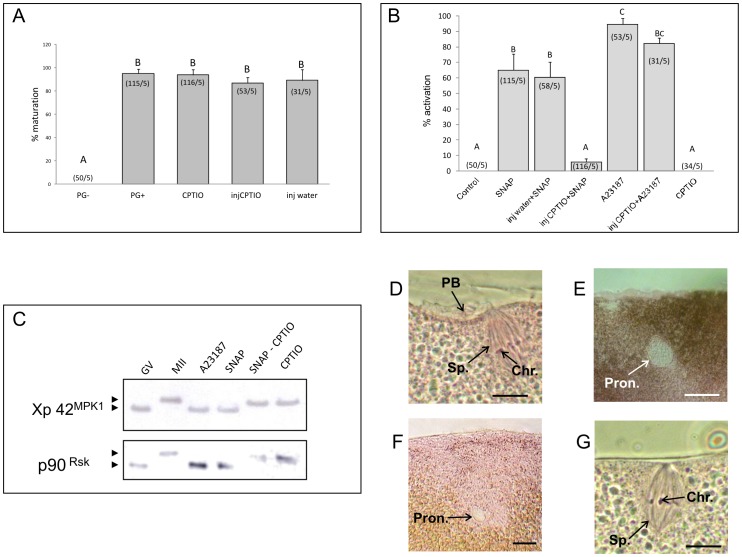
NO scavenger CPTIO does not impair maturation by, progesterone but suppresses SNAP effects on metaphase-II arrested eggs. (A) Effect of CPTIO on meiotic maturation. Histogram showing percentages of matured oocytes (with white spot) after overnight treatment with progesterone (10 µM) either in medium ND96 supplemented 10 mM CPTIO (CPTIO) or oocyte in pure ND96 injected 40 mM CPTIO (injCPTIO). Groups PG + (only progesterone), PG − (without progesterone) and inj water (progesterone and injection 15nl water) was a control of maturation and effect of microinjection. Error bands represent ± SEM values. Different superscripts indicate significant differences (P<0.05). (B) Histogram showing percentages of parthenogenetic activated oocytes after 2 hours treatment with 5 mM SNAP, 10 mM CPTIO or 10 µM A23187 injected or non-injected 40 mM NO scavenger CPTIO or water. Error bands represent ±SEM values. Different superscripts indicate significant differences (P<0.05). Data are shown as mean percentage of activation of five independent experiments. (C) Western blot analysis. After 2 hours ocytes were taken off, homogenized in lysis buffer, and immunoblotted with antibodies against Xp42^Mpk1^ and p90^Rsk^. (D) Control oocyte in metaphase II arrested oocytes exhibits typical bipolar spindle with chromosomes and the first polar body (nuclear red/picro-indigo carmine staining). (E) Activation after A23187 treatment: typical pronucleus. (F) Oocyte activated by SNAP with pronucleus. (G) Oocyte after SNAP&CPTIO treatment in metaphase II. Scale bars represent 10 µm (D,G) and 40 µm (E,F).

**Table 3:** Effect of SNAP, A23187 and SNAP+CPTIO on spindle morphogenesis and pronuclear formation following progesterone-induced maturation.

Treatment	Number of oocytes (number of females)	Metaphase spindle		Pronucleus migration		Pronucleus		No structure	
		n	%	n	%	n	%	n	%
Control	15(3)	11	73.4	0	0	0	0	4	26.6
SNAP 5 mM	35(4)	1	2.8	12	34.3	4	11.4	18	51.5
SNAP 5 mM/CPTIO 40 mM	23(3)	18	78.3	0	0	0	0	5	21.7
A23187 10 µM	18(3)	0	0	2	11.1	12	66.7	4	22.2

Calcium limited medium (120 mM NaCl, 7.5 mM KCl, 500 µM MgSO_4_, 150 µM CaCl_2_, HEPES 22.5 µM, EDTA 400 µM, pH 7.4) and calcium free medium (96 mM NaCl, 2 mM KCl, 5 mM MgCl_2_, HEPES 5 mM, EGTA 500 µM, pH 7.4) were prepared 1 hour before using. Stock solution of Ca(2+)-ionophore A23187 (Boehringer Mannheim) (100 mM), NOR5 (200 mM) (Alexis Corp.) and BAPTA-AM (100 mM) (Sigma-Aldrich) were made in DMSO and stored at −20°C. NO donors SNAP (Alexis Corp.) and SNP (Sigma-Aldrich), and also nitric oxide scavenger - CPTIO (Alexis Corp.) were prepared fresh in appropriate mediums (1 hour before using).

### Microinjections

The NO-scavenger CPTIO were micro-injected into immature oocytes, by the use of a positive displacement digital micropipette (Nichiryo) in ND96. After injection of NO-scavenger (15 nl of 40 mM stock solution), oocytes were allowed to recover for at least 1 hour and were stimulated by progesterone for overnight maturation.

**Figure 4:**
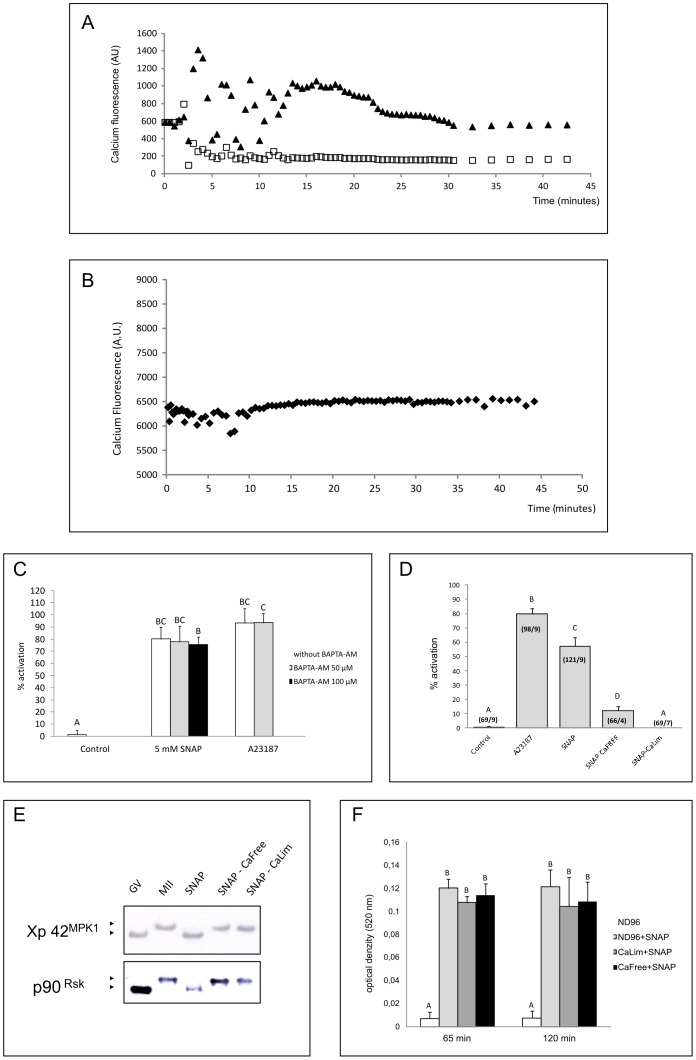
SNAP induced parthenogenetic activation is calcium-dependent. Effects of 5 mM SNAP application on mature oocyte in ND96 (black triangles) or in ND96 with 50 µM BAPTA-AM (white squares) (A) or in presence of CPTIO (B). Typical results are depicted. Fluorescence was expressed in arbitrary units and background and auto-fluorescence were subtracted. SNAP superfusion, which started at time  = 5 minutes, was responsible for a rise of intracellular calcium fluorescence. The latter was abolished by presence of CPTIO (C) Effect of calcium chelator on SNAP induced activation. Histogram showing percentages of activated oocytes after 2 hours treatment with 5 mM SNAP or 10 uM A23187 supplemented 50 µM or 100 µM BAPTA-AM. Oocyte in control group were treated for 2 hours in clear ND96. Error bands represent ±SEM values. Different superscripts indicate significant diferences (P<0.05). Data are shown as mean percentage of activation of minimally four independent experiments. (D) Percentages of parthenogenetic activated oocytes after 2 hours treatment with 5 mM SNAP in ND96 medium, calcium free medium (CaFree) and calcium limited medium (CaLim). Error bands represent ±SEM values. Different superscripts indicate significant differences (P<0.05). Data are shown as mean percentage of activation of minimally four independent experiments. (E) Western blot analysis. After 2 hours oocytes were taken off, homogenized in lysis buffer, and immunoblotted with antibodies against Xp42^Mpk1^ and p90^Rsk^. (F) Releasing of nitric oxide in calcium restricted mediums. Nitric oxide contents after SNAP treatment was determined in ND96, CaLim and CaFree mediums by colorimetric measurement of NO nitric oxide metabolites, nitrites and nitrates (NO_3_^−/^NO_2_). Each measurement was repeated three times. Different superscripts indicate significant differences (P<0.05).

### Cytological Analysis and Cortical Granule Lectins Secretion Assay

For cytological analysis, oocytes were fixed overnight in Smiths fixative, dehydrated, and embedded in paraffin. Sections (7 µm thickness) were stained with nuclear red to detect nuclei and chromosomes and with picroindigo carmine that reveals cytoplasmic structures [40].

Cortical granule lectins secretion in response to SNAP was assayed using SDS-PAGE [41]. For gel analysis (unless otherwise stated), individual oocytes were incubated in 15 µl of ND96 medium with SNAP at the concentration 5 mM. After 60 min, media were collected and added with one volume of Laemmli 2X buffer 4% beta-mercaptoethanol and incubated for 5 min at 95°C. Lectins were separated on 12.5% gels using molecular weight markers from Bio-Rad and either stained using SYPRO®Ruby.

### Electrophoresis and Western-blotting

Eggs were lysed in homogenization buffer and centrifuged for 5 min at 10 000×*g* (4°C) to eliminate yolk platelets. Supernatants were added with one volume of Laemmli 2X buffer 4% beta-mercaptoethanol, heated at 100°C for 3 min and stored at −20°C until analysis. Proteins from oocytes were separated by SDS-PAGE (15–17%) and transferred onto a nitrocellulose membrane (Hybond, Amersham Pharmacia Biotech, United Kingdom). Blots were blocked with 5% low fat dry milk and incubated with specific antibody. p90^Rsk^ was detected using the polyclonal rabbit antibody (p90^Rsk1^ C-21 sc231, Santa Cruz Biotechnology, 1/1000), Xp42^MPK1^ was detected using mouse monoclonal antibody (Erk2 D-2 sc1647, Santa Cruz Biotechnology, 1/500), Cyclin B was detected using mouse monoclonal antibody (sc-53239, Santa Cruz Biotechnology, 1/1250), anti-mos (C237, Santa Cruz Biotechnology, 1/5 000), anti-P-H3 antibodies (S10, Cell Signaling, 1/5 000) and P-Tyr15 Cdk1 using overnight incubation with mouse monoclonal antibody (tyr15 Cell, 1/10 000). Nitrocellulose membranes with bounded primary antibody were then incubated with appropriate secondary antibodies (1/2500). The signals were detected through chemiluminescent assay (ECL blotting detection kit, Amersham Pharmacia Biotech, United Kingdom).

### Immunoprecipitation

Immunoprecipitation of cdk1 was performed on batches of 30 oocytes lysed in 300 µl of buffer (50 mM HEPES pH 7.4, 500 mM NaCl, 0.05% SDS, 0.5% Triton X100, 5 mM MgCl2, 1 mg/ml bovine serum albumin, 10 µg/ml leupeptin, 10 µg/ml aprotinin, 10 µg/ml soybean trypsin inhibitor, 10 µg/ml benz-amidine, 1 mM PMSF, 1 mM sodium vanadate). After a centrifugation at 4°C for 15 min at 10,000 g, supernatants were incubated with anti-cdk1 (1∶200; Invitrogen) antibodies for 2 h at 4°C. Protein A-Sepharose beads (5 mg, Sigma) were added for 1 h at 4°C. Immune complexes were collected by centrifugation, rinsed three times, resuspended in Laemmli sample buffer, and subjected to a 10% SDS-PAGE. Immune complexes were analyzed by Western blotting using anti-cdk1 (A17, Santa Cruz Biotechnology, 1/10 000) and anti-cyclin B2 (X121.10, Santa Cruz Biotechnology, 1/5 000) antibodies and the advanced ECL detection system (Amersham Biosciences).

### Calcium Imaging

Prior imaging experiments, meiotic resumption was induced by application of 10 µM progesterone on stage VI *Xenopus* oocytes (see first section of Methods). For calcium imaging, oocytes were loaded with the calcium sensitive non-ratiometric fluorescent probe Fluo4 (acetoxy-methyl-ester derivative, Fluo4-AM, 25 µM; excitation performed at 494 nm and emission at 516 nm). The dyes were allowed at least 5 hours (Fluo4-AM) to equilibrate within the oocytes.

Fluorescence experiments were performed at room temperature (18–20°C) in ND96 solution with 5 mM SNAP. Some of these trials were conducted with BAPTA-AM, a well-known calcium chelator. Matured oocytes, at metaphase II stage, were loaded with 50 µM BATA-AM for 30 minutes [30], [42]. Acquisitions were done (every minute) on the animal hemisphere of the oocytes using an epifluorescence scanning system fitted to a Nikon microscope (Paris, France). Images were collected with Nikon® software (NIS Elements AR 3.00) and analyzed using ImageJ® software (National Institutes of Health, Bethesda, MD). Fluorescence was expressed in arbitrary units and background and auto-fluorescences were subtracted. Data obtained out of these experiments were from multiple oocytes (at least 3 batches and 3 oocytes for each).

### Measurement of Nitric Oxide Metabolites

Nitric oxide was determined by colorimetric measurement of NO nitric oxide metabolites, nitrites and nitrates (NO_3_^−/^NO_2_^−^). Nitrates were enzymatically converted by NO–reductase into nitrites. Nitrites were quantified using Griess reagents (p–aminobenzensulfonamide in 3.0 N HCl and N–(1–naftyl) ethylendiamin dichloride). The amount of nitrites was measured spectrophotometrically on microtitration plates using an ELISA plate reader Rainbow (wavelength 520 nm, Austria, SLT).

### Statistical Analysis

The number of experimental replicates is indicated in the figure legends. Data are presented as means ± S.E.M. The data were analysed by analysis of variance (Scheffés test) using statistical software (STATISTICA 8.0 StatSoft). A value of *P*<0.05 was considered as statistically significant.

## Results

### NO Donors SNAP Induces the Morphological Events of Egg Activation

The effects of nitrosothiol derivative (SNAP) – NO donor on eggs from 19 different females are summarized in Table 1. Concentrations of SNAP were chosen according to concentrations previously used in porcine oocytes in a range from 0.1 to 5.0 mM [36], [37]. No effects were observed with low concentrations of SNAP (1 mM and 2 mM) on the maintenance of the metaphase II arrest in treated eggs : matured oocytes still exhibited a white spot, where the spindle is anchored. White spot occurs during maturation when the nucleus, or germinal vesicle, moves toward the apex of cells, pushing aside the pigments. Pigment rearrangement were observed in 40% of 2.5 mM SNAP-treated eggs while 77.1% of eggs treated with 5 mM SNAP exhibited pigment rearrangement typical of those observed with control calcium ionophore A23187: the white spot disappeared while pigments were concentrated in the animal hemisphera, moving toward the apex of the cell (Fig. 1B vs 1C). Using Gundersen et al’s protocol [41], we assessed cortical granules lectins presence in 5 mM-treated eggs (Fig. 1D). SNAP induced release of cortical granule lectins in metaphase II-blocked eggs but had no effect on immature oocytes arrested in prophase I since no lectins were detected in the incubating medium (Fig. 1D). Thus, SNAP induces morphological changes related to those of cortical reaction, which are also observed for egg activation induced by calcium ionophore A23187.

Other NO donors were tested at similar concentrations (Table 2), including NOR5 and SNP, but were discarded because of (1) being respectively less potent to induce NO release (data not shown), (2) producing CN^−^ ions production, which can have secondary effects (data not shown, see also [43], [44]), (3) the toxicity of DMSO vehicle and (4) being less cell-permeant than SNAP.

### SNAP Induces MAPK Inactivation without Impairing MPF

To further characterize the effects of NO donor SNAP, western blotting analysis of meiotic key components involved in metaphase II block was performed (Fig. 2). We observed that the external signs of SNAP-induced egg activation were detected after longer time exposure in comparison to calcium ionophore (Fig. 2A) (delay 70.17+/−20.11 min). While metaphase II arrested eggs exhibited Xp42^MPK1^ and its downstream effector p90^Rsk^ under their phosphorylated and active isoforms, SNAP exposure drives the inactivation of both proteins within 30 min after the addition of the NO donor (Fig. 2B). Noticeably, Mos was not degraded and remained at constant level in this condition, in contrast to A23187-treated eggs where it is degraded after 70 minutes (Fig. 2C). MPF activity was not impaired by SNAP treatment: Cyclin level remained stable, Cdk1 was not phosphorylated on tyrosine 15 and histone H3 was phosphorylated, which attested the activity of MPF (Fig. 2C). Moreover, in order to determine if both components of MPF were still in complex, immunoprecipitation were performed using antibodies against Cdk1. Such strategy revealed that both partners were still in a complex (Fig. 2D). Control oocytes treated by Calcium Ionophore A23187 exhibited a drop in MPF activity (Cyclin B degradation, absence of phospho-Histone H3).

### SNAP Effects are Dependent upon NO Release

We investigated the effect of NO scavenger (CPTIO) microinjection on NO-induced eggs activation. To efficiently overcome the effects of NO-donor SNAP, CPTIO was added in ND96 prior to the stimulation of maturation by progesterone. NO scavenger did not impact maturation in these conditions (10 mM, Fig. 3A). When 5 mM SNAP were applied on CPTIO microinjected eggs, no morphological changes were observed (Fig. 3B) and both Xp42^MPK1^ and p90^Rsk^ remained phosphorylated (Fig. 3C). Furthermore, classical histological analysis revealed that after injection by CPTIO, SNAP-treated eggs remained in metaphase II (Table 3; Fig. 3D–G). In SNAP-treated eggs, pronuclei were observed (11.4% at membrane and 34.3% pronuclei migrated in the subcortical area, Table 3) but no metaphase spindle has been detected, suggesting that the latter has been disorganized due to NO release effects. Then, while CPTIO did not block the effects of A23187 but suppresses the effects of SNAP, both morphological and biochemical changes observed in SNAP treated oocytes have to be attributed to NO release and not to non-specific effects of SNAP itself.

### SNAP Effects Depend upon Calcium Increase

Finally we tested the hypothesis that the activation of *Xenopus* eggs via the NO-donor SNAP was mediated through calcium increase or mobilization. We observed a strong increase of intracellular calcium fluorescence induced by SNAP within the first 30 min of the treatment (Fig. 4A). This effect was specific towards NO release since CPTIO treatment completely abolished the increase observed in presence of SNAP (Fig. 4B). Treatment of oocytes with BAPTA-AM suppressed the effect of SNAP on intracellular calcium changes but BAPTA-AM alone failed to block SNAP induced cortical reaction and pigment rearrangement (Fig. 4C). We subsequently tested two different media: calcium free medium (CaFree) and calcium limited medium (CaLim), the latter being less drastic for calcium deprivation and known to prevent mechanical and spontaneous parthenogenetic activations. In our hands, both media impaired eggs activation induced by SNAP (57.2+/−5.9, 11.9+/−2.9 and 0+/−0, respectively for SNAP incubation in ND96, calcium free medium and calcium limited medium (Fig. 4D). Western blot analyses confirmed that p90^Rsk^ and Xp42^MPK1^ remained phosphorylated and under an active state after 2 hours treatment in calcium free or calcium limited medium (Fig. 4E). Releasing of NO from SNAP was similar in all three tested mediums (Fig. 4F).

## Discussion

The role of NO-dependent signaling cascade in egg activation remains unclear and is subjected to intensive research. NO donors have been considered as potential tool to promote parthenogenetic activation in different modes, such as sea urchin [33] and porcine oocytes [36], while NO may be also involved in mechanisms of fertilization in sea urchin [35] or mouse oocytes [45]. NO signaling molecules and NO levels have become issues in parthenogenetic activation and *in vitro* culture of oocytes in vertebrate’s models. Here we took advantages of the amphibian model (amenability to biochemical studies, one living cell imaging, ease of manipulation and culture independent towards follicular cells) to test the hypothesis that the NO-donor (SNAP) could induce parthenogenetic activation and exit from the metaphase II block in lower vertebrate eggs.

Several nitric oxide donors were tested: SNAP (S–nitroso-N-Acetyl-D,L-penicillamine), SNP (Sodium nitroprusside) and NOR5 (±)-2-((E)-4-Ethyl-3[(Z)-hydroxyimino]6-methyl-5-nitro-heptenyl)-3-pyridinecarboxamide). SNP and NOR5 were rapidly discarded, and SNAP was chosen due to its effects on *Xenopus* eggs and its ease of use in our context. NOR5 and SNP did not exert any effects on metaphase II block (data not shown); SNP is regarded as less potent than SNAP to induce NO release ( [46]; M. Jeseta, personal observations), has to be exposed to light for nitric oxide release [47] and finally may produce cyanide CN- ions following its breakdown [46], [48]. The absence of effects of NOR5 at the concentrations used (2 mM) may be due to the fact that this compound is believed to be less permeant than SNAP. Its injection into *Xenopus* eggs was not considered because of NOR5 being solubilized in DMSO, which could exert itself deleterious effects on metaphase II block [23].

SNAP is a stable analogous of endogenous S-nitrosothiols, and NO is released from it by endogenous enzymes after the penetration of SNAP into the cell [49]–[50]. On a physiological note, endogenous NO may be produced by three different isoforms of NOS. The presence of eNOS and iNOS isoforms was observed in fully-grown rat [51], mouse [52], porcine [53], [54] cattle oocytes [55]. In *Xenopus*, only nNOS was detected [56]. Similarly to porcine eggs, SNAP long time exposures were requested to observe external signs of parthenogenetic activation, as attested by pigment rearrangement [36]. Eggs permeability to NO-donors or NO releasing rates may be determining and limiting factors in the slow dynamic of cortical reaction observed in these models. In our hands, SNAP induced cortical granules exocytosis, in contrast to porcine oocytes treated with NO-donor, which did not exhibit the exocytosis of cortical granules [36].

Because cortical reactions can be considered as deconnected from metaphase II exit *per se*, we checked for the activity and activation status of MPF and MAPK. MAPK and its downstream effector p90rsk were dephosphorylated within 30 minutes post-addition of SNAP whereas MPF remained under an active profile (tyrosine 15 unphosphorylated and maintenance of high levels of Cyclin B). Inhibition of MPF activity could have been driven in these conditions by the dissociation of Cdk1 from its regulatory subunit. The Cdk1-Cyclin B dissociation is sufficient to dramatically impair MPF activity [57], [58]. Nevertheless, in our hands, SNAP exposure failed to promote the disassembly of the MPF, because both partners of the heterodimer, Cdk1 and Cyclcin B, were immunoprecipitated together. Finally, maintenance of MPF activity was firmly suggested by the phosphorylation of Histone H3 and the absence of Mos degradation. Indeed, Mos degradation is prevented by the activity of MPF, which phosphorylates Mos on its residue serine 3, thus protecting the protein from its degradation [59]. Then, MAPK activity declines while MPF remains active in SNAP-treated eggs. Interestingly, the decrease of MAPK activity was correlated to the disorganization of spindle in SNAP-treated eggs. Pigments trails from pronucleus migration and pronuclei were observed in these conditions. Similarly, interphase transition has been reported in metaphase II-blocked porcine oocytes treated with MEK inhibitor U0126 [60], [61].

CPTIO was successfully used in different cells types, including gametes and embryos, to block NO-release [62]–[64]. It has been previously reported differences in mammalian oocytes regarding sensitivity on nitric oxide during maturation. High concentration of NO donors supress nuclear maturation in bovine [65] and porcine oocyte [66]. However low doses of NO donor stimulate nuclear maturation in mouse [39] or cattle oocyte [65]. On the oher hand NOS inhibitors block nuclear maturation in pig [54] or mouse oocyte [8]. In our conditions, NO-scavenging did not appear to impair the progression of M-phase entry and maturation in *Xenopus* oocytes, because CPTIO-treated oocytes treated with progesterone resumed and completed meiosis like control ones (Fig. 3A). These results indicate that the role of NO for regulation in meiotic maturation is not crucial for *Xenopus* oocytes maturation, in contrast to mammalian oocytes. Nevertheless, microinjection of NO scavenger blocked the effects of SNAP on eggs, enabling us to discard the hypothesis that SNAP effects are non specific: MAPK inactivation, spindle disorganization and pronuclei formation induced by SNAP were indeed severely impaired by CPTIO.

From literature, calcium- and NO-dependent signaling pathways are clearly interconnected in oocytes and eggs with calcium playing a pivotal and main role [33], [35], [37]. The NO-dependent signaling cascade is regulated by calcium. The important role of calcium for NO induced activation was also confirmed by our study, which demonstrated that NO-induced parthenogenetic activation is impaired in calcium limited or calcium free media. Production of NO in the cell depends on activation of NOS by Calmodulin, which is itself activated by calcium ions [1], [4]. Mainly, NO influences the levels of intracellular calcium through the regulation of calcium ion channels and pumps, which modulate the influx and efflux of calcium between the cell and extracellular space [67]. NO could also regulate the mobilization of calcium ions from their intracellular stores. Calcium from this source is released through either ryanodine (RYR) or inositol triphosphate receptors (IP_3_R) [68]. Interestingly NO-dependent activation of pig oocytes depends only on mobilization of intracellular calcium stores from RYRs. The mobilization of endogenous calcium stores through IP_3_R is not necessary for SNAP parthenogenetic activation in porcine [37].

In *Xenopus laevis* oocytes the study of the elementary events of calcium signalling is facilitated by the lack of ER calcium-release channels (e.g., ryanodine receptors (RYRs) and cADP-ribose receptors) other than IP_3_Rs [30]. In fact, RYR are poorly expressed and located near nucleus in *Xenopus* oocytes. Therefore, the role of RYR is rather considered as modest for endogenous calcium release mechanisms in contrast to pig oocytes [69], [70]. Our results suggest that nitric oxide specifically induces parthenogenetic activation in Xenopus laevis eggs, through a calcium dependent mechanism, though the origin of the calcium changes in our context remains to be determined. Cell cycle control and progression, such as maturation and activation processes, imply different ionic modifications, including particularly calcium [71]. Calcium sources could be differently mobilized and result in dramatic as well as discrete changes, at local levels. For example, it is well known that, in mature oocyte, IP3 receptors are clustering at the membrane of the endoplasmic reticulum [72], [73]. This could be responsible for calcium variations in restricted areas – microdomains - where calcium concentration could rise but may be also difficult to detect and measure by oocyte imaging.

Inactivation of MAPK at metaphase II, while MPF is still active, offers an atypical playground to unravel the effects of NO on cell cycle regulation mechanisms at fertilization. In one hand, NO induced MAPK inactivation and cortical granule exocytosis through a calcium increase but on another hand, the increase in calcium failed to inactivate MPF. Why does not the calcium increase drive the inactivation of MPF? Nitrosylation has been reported in other cellular models to impair CaMKII activity [74], [75]. Then, NO increase may lead to the inactivation of CaMKII and prevent the activation of APC, which is necessary for MPF inactivation and cyclin B degradation. Because calcium limited and calcium free media prevented the inactivation of MAPK induced by SNAP, a calcium-dependent event is involved in the inactivation of MAPK, and not nitrosylation itself. It was previously reported that nitric oxide induced activation of mammalian oocytes is stimulated by alternative signaling pathways, which are not involved in oocyte activation after treatment by traditional activating protocols (i.e. by calcium ionophore). Thus, mechanism of nitric oxide effect on matured oocytes remains to be determined. Further studies are also requested to clarify the role of nitric oxide in oocyte biology.
